# Targeted delivery to inflammatory monocytes for efficient RNAi-mediated immuno-intervention in auto-immune arthritis

**DOI:** 10.1186/1479-5876-9-S2-P38

**Published:** 2011-11-23

**Authors:** Jessy Présumey, Gabriel Courties, Louis-Marie Charbonnier, Virginie Escriou, Daniel Scherman, Yves-Marie Pers, Pascale Louis-Plence, Diego Kyburz, Steffen Gay, Christian Jorgensen, Florence Apparailly

**Affiliations:** 1 Inserm, U 844, CHU Saint Eloi University Hospital, Montpellier, France; 2 University of Medicine, Montpellier, France; 3 Inserm, U 1022, UFR des Sciences Pharmaceutiques et Biologiques, Paris, France; 4 CNRS, UMR8151, Paris, France; 5 University of Pharmacy Paris Descartes, Laboratoire de Pharmacologie Chimique, Génétique et Imagerie, Paris, France; 6 Ecole Nationale Supérieure de Chimie de Paris, Paris, France; 7 CHU Lapeyronie University Hospital, Clinical Dept. for Osteoarticular Diseases, Montpellier, France; 8 Center of Experimental Rheumatology, University Hospital Zurich and Zurich Center of Integrative Human Physiology, Zurich, Switzerland

## 

Inflammatory mouse Ly6C^high^ monocyte subset and its human counterpart, defined as CD14^+^ CD16^-^, represent a valuable cellular target for innovative immunotherapeutic strategies against immune-mediated inflammatory disorders (IMID). However, delivery systems able to differentially target both subsets in vivo are still missing as well as demonstration for efficient immuno-modulation. The present work aims at providing evidences for the selective delivery of a siRNA-containing lipid formulation to the Ly-6C^high^ monocyte population and at evaluating the therapeutic potential of targeting this subset as well as their human counterpart for immuno-intervention in a prototype IMID like rheumatoid arthritis (RA). The pre-B-cell colony enhancing factor (PBEF/visfatin/Nampt) is an essential enzyme in the NAD biosynthetic pathway that exerts a key role in the persistence of inflammation through the induction of the expression of the TNF-α and IL-6 pro-inflammatory cytokines and is highly expressed in patients with a variety of IMID. Mice with collagen-induced arthritis (CIA) display Ly-6C^high^ monocytosis in the circulation that infiltrate into the inflamed joints. The systemic delivery of siRNAs formulated with the cationic liposome DMAPAP provides specific and functional down-regulation of PBEF within inflammatory monocytes. Moreover, decreased production of the PBEF-induced pro-inflammatory cytokines TNF-α and IL-6 was evidenced in both mouse and human inflammatory monocytes. PBEF gene silencing within Ly-6C^high^ monocytes resulted in reduced disease severity in mice with CIA, associated with an overall systemic immuno-modulation of the effector T cell balance. These results identify PBEF as a critical target to modulate autoimmune responses and inflammation in arthritis and provide novel evidence that silencing of a master gene within inflammatory monocytes is a promising strategy for future therapeutic intervention in the context of IMID.

